# Effect of exercise intervention on social distance in middle-aged and elderly patients with chronic low back pain

**DOI:** 10.3389/fnagi.2022.976164

**Published:** 2022-08-22

**Authors:** Lin-Man Weng, Rui Wang, Qi-Hao Yang, Tian-Tian Chang, Cheng-Cheng Wu, Wen-Long Li, Shu-Hao Du, Yu-Chen Wang, Xue-Qiang Wang

**Affiliations:** ^1^Department of Sport Rehabilitation, Shanghai University of Sport, Shanghai, China; ^2^College of Chinese Wushu, Shanghai University of Sport, Shanghai, China; ^3^Department of Rehabilitation Medicine, Shanghai Shangti Orthopedic Hospital, Shanghai, China; ^4^Shanghai Key Lab of Human Performance, Shanghai University of Sport, Shanghai, China

**Keywords:** social distance, chronic low back pain, exercise therapy, stop-distance, personal space

## Abstract

**Background:**

Increased social distance is one of the manifestations of social impairment. Chronic low back pain (CLBP) is one of factors associated with increased social distance and social withdrawal. Exercise therapy is an effective means to social impairment. However, whether exercise could reduce social distance in patients with CLBP remains unknown. This study aimed to investigate the effect of exercise on social distance in middle-aged and elderly patients with CLBP.

**Methods:**

The longitudinal intervention recruited 29 middle-aged and elderly patients with CLBP from various communities in Yangpu District, Shanghai, China. The participants received exercise intervention for 8 weeks. The assessments were conducted before and after the intervention, including social distance, pain intensity, unpleasantness of pain, Roland-Morris Questionnaire (RMDQ), Self-Rating Anxiety Scale (SAS), and Self-Rating Depression Scale (SDS). Intention to treat analysis was performed.

**Results:**

After the 8-week exercise intervention, the social distance of patients with CLBP was shorter than that before intervention and showed significant difference (*p* < 0.05). The scores of pain intensity, unpleasantness of pain, RMDQ, SAS, and SDS also decreased and were significantly different between pre- and post-intervention (*p* < 0.05). In addition, the social distance, pain intensity, unpleasantness of pain, RMDQ, SAS, and SDS scores of the moderate CLBP group decreased more after the intervention compared with those of the mild CLBP group.

**Conclusion:**

The 8-week exercise intervention cannot only shorten the social distance in middle-aged and elderly patients with CLBP but also relieve pain, disability, and negative emotions.

## Introduction

Low back pain (LBP) is a common symptom experienced by people of almost all ages, and it is one of the main reasons for seeking medical health care (Hartvigsen et al., 2018). The lifetime prevalence of LBP is approximately 84%, and the prevalence of chronic LBP (CLBP) is approximately 23% (Balagué et al., 2012). CLBP can lead to physical, psychological, and social dysfunction. Along with pain and disability, patients with CLBP often experience depression and anxiety and have a negative impact on social, entertainment, and work life. Our previous study has also found that patients with CLBP exhibit social withdrawal and great social distance (Weng et al., 2021). In addition, CLBP causes a huge economic burden to families and society. In the United States, the total cost of LBP is more than $100 billion a year, two-thirds of which is the indirect cost of lost work or reduced productivity (Katz, 2006; Deyo et al., 2014).

Social distance is mediated by comfort or discomfort resulting from the distance between individuals and others. Appropriate social distance is the basis for establishing effective communication and good interpersonal relationships, while too short social distance will cause discomfort and anxiety (Gessaroli et al., 2013; Perry et al., 2016). The increase of social distance is one of the manifestations of social withdrawal, which is caused by poor mental health (Simon and Walker, 2018; Achterbergh et al., 2020).

Most guidelines recommend exercise therapy as an intervention for CLBP (Chou et al., 2017; Qaseem et al., 2017). Exercise is more effective than no-exercise intervention for CLBP (Hayden et al., 2005; van Middelkoop et al., 2010; Searle et al., 2015). Passive therapy alone (such as ultrasound, cold therapy, heat therapy, massage) offers limited improvement of the pain and physical function in patients with CLBP (Furlan et al., 2002; French et al., 2006; Ebadi et al., 2020; Owen et al., 2020). Numerous studies suggested that exercise therapy can relieve pain, improve back muscle strength, enhance physical function, and prompt mental health (Searle et al., 2015; Owen et al., 2020; Peng et al., 2022; Wu et al., 2022). Furthermore, exercise can improve social withdrawal but is mainly concentrated in diseases involving severely impaired social function (such as mental illness) (Richardson et al., 2005; Firth et al., 2015; Kimhy et al., 2016; van der Stouwe et al., 2018).

To our knowledge, a limited number of studies explored the benefits of exercise on social psychology in patients with CLBP. Whether exercise can improve social withdrawal and reduce social distance in patients with CLBP remains unknown. Thus, we conducted a longitudinal intervention study to investigate the effect of exercise on social distance in patients with CLBP over an 8-week intervention period.

## Materials and methods

### Study design

We conducted an 8-week exercise intervention to explore the effect of exercise on social distance in patients with CLBP. Two rounds of assessments, including those for social distance task, pain intensity, unpleasantness of pain, Roland-Morris Questionnaire (RMDQ), Self-Rating Anxiety Scale (SAS), and Self-Rating Depression Scale (SDS) were completed pre- and post- intervention.

### Participants

The effect size was calculated to be 0.67 in accordance with the study by Cho (2014). After G*Power calculation (two tails, α = 0.05, power = 0.80), the sample size was 20. Considering that the turnover rate of 20%, 25 subjects were required for this study. Finally, 29 middle-aged and elderly patients with CLBP were recruited from various communities in Yangpu District, Shanghai, China. The inclusion criteria were as follows: (1) aged 50–75 years old; (2) with LBP lasting for at least 12 weeks; (3) with maximum pain intensity of at least 3 on the Numerical Rating Scale (NRS); (4) no pain in other parts of the body except LBP; (5) no cognitive impairment and can understand the experimental content. The exclusion criteria were as follows: (1) Specific LBP caused by definite tissue structure or pathology, such as intervertebral disc (or spinal canal) disease, tumor, visceral disease, spinal disease, or injury. All subjects met the inclusion and exclusion criteria, voluntarily attended to the study, and signed an informed consent. The study was approved by the Ethics Committee of Shanghai University of Sport.

### Outcome measures

Assessments were performed at baseline and after the 8-week exercise intervention, including social distance, pain intensity, disability, unpleasantness of pain, anxiety, and depression.

Social distance under various experimental conditions was measured by a stop-distance paradigm with high reliability and validity (Kennedy et al., 2009; Lough et al., 2016). It includes comfortable distance and uncomfortable distance when the subject actively approaches an experimenter (consistent or inconsistent with the subject’s gender) or when is approached. A digital laser measurer (Bosch GLM 30C) was used to measure the true distance under different experimental conditions. The NRS was applied to measure the maximum pain intensity (NRS_max_), average pain intensity (NRS_avg_), pain intensity at rest (NRS_rest_), and pain intensity at the moment (NRS_now_) during the last 3 days. The NRS is an 11-grade rating scale: 0, “no pain at all”; 1–3, “mild pain”; 4–6, “moderate pain”; 7–9, “severe pain”; 10, “maximum pain.” This scale has good validity and reliability (Bendinger and Plunkett, 2016; Yao et al., 2020). Following international consensus, a 2-point decrease for NRS was considered a minimal clinically important difference (MCID) (Ostelo et al., 2008). LBP-related disability was assessed with the 24-item RMDQ, which yields scores ranging from 0 to 24. A high score indicates a serious disability. Following international consensus, a 2-point decrease for RMDQ was considered a MCID (Ostelo et al., 2008). The NRS also measures the unpleasantness of pain (“0” means “no pain-induced unpleasantness at all,” and “10” means “maximum pain-induced unpleasantness”), including the average unpleasantness of pain (NRS_unavg_) and unpleasantness of pain at the moment (NRS_unnow_) during the last 3 days. The levels of anxiety and depression were evaluated by the 20-item SAS and the 20-item SDS, respectively. The total scores of both scales are 20–80. A high score means serious anxiety or depression (Yu et al., 2012).

### Social distance task

**FIGURE 1:**
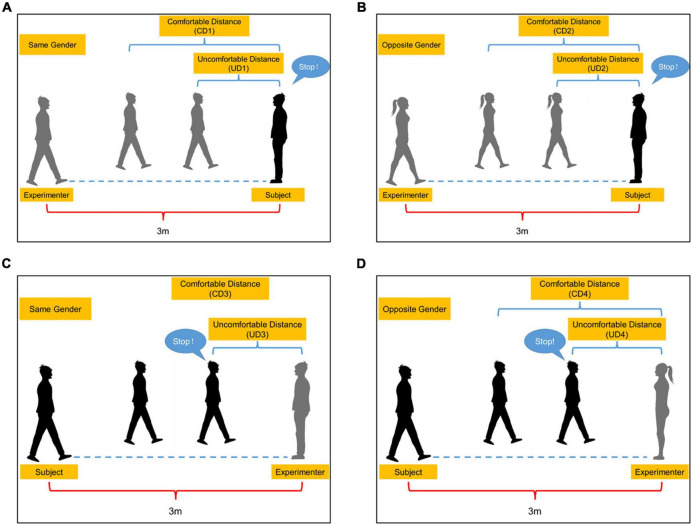
Social distance task. **(A)** The subject (black) is approached by the experimenter (gray) of the same gender. **(B)** The subject (black) is approached by the experimenter (gray) of the opposite gender. **(C)** The subject (black) walks toward the experimenter (gray) of the same gender. **(D)** The subject (black) walks toward the experimenter (gray) of the opposite gender. CD1, The comfortable distance when the subject is approached by the experimenter of the same gender; CD2, The comfortable distance when the subject is approached by the experimenter of the opposite gender; CD3, The comfortable distance when the subject walks toward the experimenter of the same gender; CD4, The comfortable distance when the subject walks toward the experimenter of the opposite gender; UD1, The uncomfortable distance when the subject is approached by the experimenter of the same gender; UD2, The uncomfortable distance when the subject is approached by the experimenter of the opposite gender; UD3, The uncomfortable distance when the subject walks toward the experimenter of the same gender; UD4, The uncomfortable distance when the subject walks toward the experimenter of the opposite gender.

A stop-distance paradigm has been widely applied in the studies of detecting individual social distance (Kennedy et al., 2009; Lough et al., 2016). The starting distance of the task was 3 m, and the subjects and experimenters were unfamiliar with each other. Task 1: The experimenter (same gender as the subject) walked toward the subject who was standing still at a natural pace. They looked into each other’s eyes. The experimenter stopped walking when he reached a distance that the subject normally keeps from strangers. This distance was the comfortable distance (CD1, Figure 1A). Thereafter, the experimenter continued walking and stopped when he reached a distance at which the subject felt uncomfortable. This distance was the uncomfortable distance (UD1, Figure 1A). Task 2 (like Task 1): The subject walked toward the experimenter and stopped at a comfortable distance (CD3, UD3; Figure 1C). After the bidirectional tests, the process was repeated, but with the experimenter of the opposite gender (Task 3 and 4, Figures 1B,D). The distance was measured with a digital laser measurer (Bosch GLM 30C). Each task was performed thrice, and the average was considered the outcome.

### Intervention

Tai Chi was selected as the intervention method of the study. The clinical practice guideline from the American College of Physicians strongly recommends Tai Chi as a non-invasive treatment for CLBP (Qaseem et al., 2017). Tai Chi moves slowly, gently, and rhythmically, with smooth circular motion of the upper limbs and continuous weight shifting of the low limbs (Peng, 2012). Previous studies have confirmed that Tai Chi is a safe and effective exercise. It cannot only improve physical functions, such as by alleviating LBP, enhancing muscle strength and endurance, and promoting core stability, but also improve mental health, including through alleviating depression and anxiety and helping a person to relax (Hall et al., 2009; Cho, 2014).

The intervention consisted of 8 Yang-Style Tai Chi movements: (1) Part the Wild Horse’s Mane; (2) Fair Lady Works at Shuttles; (3) Brush Knee and Twist Step; (4) Pull, Block and Pound; (5) Grasp the Peacock’s Tail; (6) Step Back and Whirl Arms on Both Sides; (7) Golden Rooster Stands on One leg; (8) Cloudy Hands (Wang et al., 2022). It adopted group training. Tai Chi was carried out by a trained professional Tai Chi coach to give instruction and correct the wrong actions in time. The program lasted for 8 weeks, with a session lasting for 60 min and being conducted twice a week. An intervention session included 10 min warm-up exercise, 40 min Tai Chi, and 10 min relaxation training. The exercise intensity of the subjects was controlled at 3–5 in the Borg Scale, accounting for 40–60% of their maximum heart rate. In the study, the first to third weeks were allotted for learning and mastery of the single movements of Tai Chi, whereas the fourth to eighth weeks involved repetitive practicing and consolidation of the integrated movements.

### Statistical analysis

All results were analyzed by the IBM SPSS Statistics 26. Quantitative variables were presented as mean ± standard deviation ($\bar{x}\pm s$). All data were tested for normality by Shapiro-Wilk test and normal transformation when necessary. The parameter test was used for the data conformation to normal distribution, and non-parametric test was used for data with severely skewed distribution and cannot be converted to a normal one. *p* < 0.05 was considered statistically significant.

Based on the average pain intensity, we divided the data into three groups, namely, overall, moderate (NRS_avg_ > 3), and mild (NRS_avg_ ≤ 3) CLBP group. The inter-group comparison of baseline data, the result of pre-intervention, and post-intervention—pre-intervention were compared by the independent sample *t*-test, Mann-Whitney *U*-test and Chi-Square test. Paired sample *t*-test or Wilcoxon signed-rank test was selected to detect the intra-group differences pre- and post-intervention. All the results were analyzed by intention-to-treat, and the method of regression estimation was performed to fill in the missing values. Subjects who had an attendance rate of less than 75% or failed to attend the assessment were considered to have been dropped out.

## Results

Of the 49 middle-aged and elderly subjected recruited, 12 did not meet the inclusion criteria, 5 declined to participate, 3 had other reasons for inability to participate, and finally 29 met the inclusion criteria and received 8-week exercise intervention. The flow of subjects through the study was shown in Supplementary Figure 1. Three subjects participated in Tai Chi courses less than 12 times across the study period (Supplementary Figure 1). Overall, the mean age was 65.90 (4.97) years, 9 subjects were men (31.03%), and 20 were women (68.97%). A total of 13 subjects were included in the moderate CLBP group, with an average age of 67.31 (4.35) years. Exactly 16 subjects were included in the mild CLBP group, with an average age of 64.75 (5.29) years. No significant difference was observed in baseline the information between the moderate and mild CLBP groups (*p* > 0.05, Table 1).

**TABLE 1:** Baseline demographic and clinical characteristics.

	Group, mean (*SD*)			*p*
	
	Overall (*n* = 29)	Moderate (*n* = 13)	Mild (*n* = 16)	
Male, *n* (%)	9 (31.03%)	4 (30.77%)	5 (31.25%)	0.978
Age, years	65.90 (4.97)	67.31 (4.35)	64.75 (5.29)	0.186
Height, cm	161.63 (7.62)	163.12 (7.91)	160.43 (7.42)	0.353
Weight, kg	64.62 (11.43)	64.02 (7.32)	65.12 (14.15)	0.801
BMI (kg/m^2^)	24.62 (3.18)	24.03 (1.86)	25.10 (3.94)	0.380
Smoking, *n* (%)	4 (13.79%)	1 (7.69%)	3 (18.75%)	0.390
Drinking, *n* (%)	3 (10.34%)	1 (7.69%)	2 (12.50%)	0.672
Education, *n* (%)				0.214
Primary school or below	1 (3.45%)	/	1 (6.25%)	NA
Junior high school	10 (34.48%)	7 (53.85%)	3 (18.75%)	NA
Senior high school	14 (48.28%)	5 (38.46%)	9 (56.25%)	NA
College or above	4 (13.79%)	1 (7.69%)	3 (18.75%)	NA
Course of CLBP, years	11.88 (11.14)	12.73 (13.29)	11.20 (9.45)	0.982
Types of pain				0.422
Continuous	3 (10.34%)	2 (15.38%)	1 (6.25%)	NA
Intermittent	26 (89.66%)	13 (84.62%)	15 (93.75%)	NA
Nature of pain				0.352
Aching pain	14 (48.28%)	4 (30.77%)	10 (65.50%)	NA
Bursting pain	2 (6.90%)	2 (15.38%)	/	NA
Prickling pain	2 (6.90%)	1 (7.69%)	1 (6.25%)	NA
Radiant pain	2 (6.90%)	1 (7.69%)	1 (6.25%)	NA
Mixed pain	9 (31.03%)	5 (38.46%)	4 (25.00%)	NA
SAD	7.38 (5.18)	6.38 (4.61)	8.19 (5.61)	0.361
IPAQ				NA
Total physical activity per week (MET-min/w)	4415.10 (2796.05)	4120.39 (2991.87)	4654.54 (2701.10)	0.599
Physical activity level				0.730
Low	/	/	/	NA
Medium	8 (27.59%)	4 (30.77%)	4 (25.00%)	NA
High	21 (72.41%)	9 (69.23%)	12 (75.00%)	NA

BMI, body mass index; CLBP, chronic low back pain; SAD, Social Avoidance and Distress Scale; IPAQ, International Physical Activity Questionnaire.

### Social distance outcomes

**FIGURE 2:**
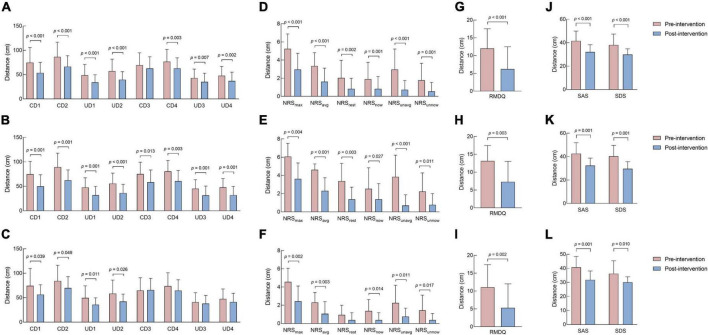
Difference in social distance, pain intensity and unpleasantness, disability, and negative emotion outcomes before and after intervention. **(A)** Difference in social distance outcomes in the overall CLBP group before and after intervention. **(B)** Difference in social distance outcomes in the moderate CLBP group before and after intervention. **(C)** Difference in social distance outcomes in the mild CLBP group before and after intervention. **(D)** Difference in pain intensity and unpleasantness outcomes in the overall CLBP group before and after intervention. **(E)** Difference in pain intensity and unpleasantness outcomes in the moderate CLBP group before and after intervention. **(F)** Difference in pain intensity and unpleasantness outcomes in the mild CLBP group before and after intervention. **(G)** Difference in disability outcomes in the overall CLBP group before and after intervention. **(H)** Difference in disability outcomes in the moderate CLBP group before and after intervention. **(I)** Difference in disability outcomes in the mild CLBP group before and after intervention. **(J)** Difference in negative emotion outcomes in the overall CLBP group before and after intervention. **(K)** Difference in negative emotion outcomes in the moderate CLBP group before and after intervention. **(L)** Difference in negative emotion outcomes in the mild CLBP group before and after intervention. CD1, The comfortable distance when the subject is approached by the experimenter of the same gender; CD2, The comfortable distance when the subject is approached by the experimenter of the opposite gender; CD3, The comfortable distance when the subject walks toward the experimenter of the same gender; CD4, The comfortable distance when the subject walks toward the experimenter of the opposite gender; UD1, The uncomfortable distance when the subject is approached by the experimenter of the same gender; UD2, The uncomfortable distance when the subject is approached by the experimenter of the opposite gender; UD3, The uncomfortable distance when the subject walks toward the experimenter of the same gender; UD4, The uncomfortable distance when the subject walks toward the experimenter of the opposite gender. NRS_max_, the maximum pain intensity during the last 3 days; NRS_avg_, the average pain intensity during the last 3 days; NRS_rest_, the pain intensity at rest during the last 3 days; NRS_now_, the pain intensity at the moment during the last 3 days; NRS_unavg_, the average unpleasantness of pain during the last 3 days; NRS_unnow_, the unpleasantness of pain at the moment during the last 3 days; RMDQ, Roland-Morris Questionnaire; SAS, Self-Rating Anxiety Scale; SDS, Self-Rating Depression Scale.

All data on social distance are presented in Figure 2 and Table 2. Before the intervention, no significant difference was recorded in each distance between the moderate and mild CLBP groups (*p* > 0.05, Table 2). Figures 2A–C shows that the distances of each group in post-intervention were shorter than those in pre-intervention. A significant difference was observed in CD1, CD2, UD1, UD2, CD4, UD3, and UD4 of the overall CLBP group, all distances of the moderate CLBP group, and CD1, CD2, UD1, and UD2 of the mild CLBP group between pre- and post-intervention (*p* < 0.05). Compared with the mild CLBP group, the moderate CLBP group had more marked changes, and the inter-group differences of post-pre CD1 (difference, –6.91; 95% confidence interval (CI): –31.28 to 17.47), CD3 (difference, –17.53; 95% CI: –34.62 to –0.43), and UD3 (difference, –10.77; 95% CI: –21.11 to –0.44) were statistically significant (*p* < 0.05) (Table 2).

**TABLE 2:** Social distance outcomes after 8-week exercise intervention.

	Group, mean (*SD*)				*p*
	
	Overall (*n* = 29)	Moderate (*n* = 13)	Mild (*n* = 16)	Between-group difference (95% CI)	
**CD1** (cm)					
Pre-intervention	74.63 (31.02)	74.83 (26.13)	74.46 (35.35)	0.38 (–23.82, 24.58)	0.770
Post-pre intervention	–21.08 (31.43)	–24.89 (19.19)	–17.98 (39.08)	–6.91 (–31.28, 17.47)	0.020*
**CD2** (cm)					
Pre-intervention	86.56 (30.14)	89.34 (28.64)	82.29 (32.05)	5.05 (–18.38, 28.48)	0.560
Post-pre intervention	–20.15 (27.18)	–27.08 (21.30)	–14.52 (30.65)	–12.55 (–33.17, 8.06)	0.054
**UD1** (cm)					
Pre-intervention	48.83 (22.28)	47.87 (19.58)	49.62 (24.87)	–1.75 (–19.12, 15.63)	0.838
Post-pre intervention	–14.84 (18.89)	–16.07 (14.26)	–13.85 (22.38)	–2.23 (–16.93, 12.48)	0.161
**UD2** (cm)					
Pre-intervention	57.22 (24.62)	55.64 (21.45)	58.50 (27.55)	–2.86 (–22.03, 16.31)	0.762
Post-pre intervention	–17.69 (21.16)	–19.47 (14.18)	–16.25 (25.88)	–3.23 (–19.69, 13.23)	0.125
**CD3** (cm)					
Pre-intervention	69.49 (25.26)	75.21 (24.06)	64.84 (26.02)	10.36 (–8.92, 29.65)	0.220
Post-pre intervention	–6.96 (23.64)	–16.63 (20.67)	0.90 (23.55)	–17.53 (–34.62, –0.43)	0.045*
**CD4** (cm)					
Pre-intervention	76.85 (24.95)	80.50 (22.50)	73.87 (27.14)	6.63 (–12.66, 25.92)	0.487
Post-pre intervention	–14.10 (23.61)	–19.97 (19.81)	–9.33 (25.93)	–10.64 (–28.58, 7.29)	0.066
**UD3** (cm)					
Pre-intervention	42.92 (18.53)	45.32 (18.10)	40.98 (19.24)	4.34 (–10.02, 18.70)	0.540
Post-pre intervention	–7.80 (14.32)	–13.74 (10.80)	–2.97 (15.31)	–10.77 (–21.11, 0.44)	0.042*
**UD4** (cm)					
Pre-intervention	47.75 (19.01)	48.00 (17.98)	47.55 (20.39)	0.45 (–14.38, 15.27)	0.951
Post-pre intervention	–10.63 (16.55)	–15.90 (12.80)	–6.34 (18.34)	–9.56 (–21.90, 2.79)	0.124
**NRS_max_**					
Pre-intervention	5.24 (1.64)	6.08 (1.44)	4.56 (1.50)	1.51 (0.38, 2.65)	0.011*
Post-pre intervention	–2.28 (2.09)	–2.46 (1.94)	–2.12 (2.25)	–0.34 (–1.96, 1.29)	0.372
**NRS_avg_**					
Pre-intervention	3.34 (1.47)	4.62 (0.65)	2.31 (1.08)	2.30 (1.60, 3.00)	<0.001**
Post-pre intervention	–1.72 (1.44)	–2.31 (1.44)	–1.25 (1.29)	–1.06 (–2.10, –0.02)	0.051
**NRS_rest_**					
Pre-intervention	2.03 (1.94)	3.38 (1.94)	0.94 (1.06)	2.45 (1.29, 3.61)	0.001**
Post-pre intervention	–1.21 (1.76)	–2.00 (1.96)	–0.56 (1.32)	–1.44 (–2.69, –0.19)	0.047*
**NRS_now_**					
Pre-intervention	1.90 (1.86)	2.54 (2.30)	1.38 (1.26)	1.16 (–0.21, 2.54)	0.160
Post-pre intervention	–1.07 (1.31)	–1.15 (1.46)	–1.00 (1.21)	–0.15 (–1.17, 0.86)	0.789
**NRS_unavg_**					
Pre-intervention	2.97 (2.26)	3.85 (2.38)	2.25 (1.95)	1.60 (–0.05, 3.24)	0.057
Post-pre intervention	–2.24 (2.29)	–3.15 (2.34)	–1.50 (2.03)	–1.65 (–3.32, 0.01)	0.052
**NRS_unnow_**					
Pre-intervention	1.79 (1.86)	2.23 (2.05)	1.44 (1.67)	0.79 (–0.62, 2.21)	0.326
Post-pre intervention	–1.24 (1.57)	–1.46 (1.51)	–1.06 (1.65)	–0.40 (–1.61, 0.81)	0.418
**RMDQ**					
Pre-intervention	12.00 (5.54)	13.15 (4.34)	11.06 (6.34)	2.09 (–2.15, 6.34)	0.291
Post-pre intervention	–5.79 (5.92)	–5.85 (5.73)	–5.75 (6.27)	–0.10 (–4.72, 4.53)	0.966
**SAS**					
Pre-intervention	41.48 (8.44)	42.54 (9.36)	40.63 (7.82)	1.91 (–4.63, 8.46)	0.553
Post-pre intervention	–9.48 (8.47)	–10.23 (8.19)	–8.87 (8.91)	–1.36 (–7.94, 5.23)	0.676
**SDS**					
Pre-intervention	38.03 (9.32)	40.31 (9.37)	36.19 (9.16)	4.12 (–2.97, 11.21)	0.186
Post-pre intervention	–8.17 (9.18)	–10.69 (8.42)	–6.12 (9.52)	–4.57 (–11.50, 2.37)	0.091

CD1, The comfortable distance when the subject is approached by the experimenter of the same gender; CD2, The comfortable distance when the subject is approached by the experimenter of the opposite gender; CD3, The comfortable distance when the subject walks toward the experimenter of the same gender; CD4, The comfortable distance when the subject walks toward the experimenter of the opposite gender; UD1, The uncomfortable distance when the subject is approached by the experimenter of the same gender; UD2, The uncomfortable distance when the subject is approached by the experimenter of the opposite gender; UD3, The uncomfortable distance when the subject walks toward the experimenter of the same gender; UD4, The uncomfortable distance when the subject walks toward the experimenter of the opposite gender. NRS_max_, the maximum pain intensity during the last 3 days; NRS_avg_, the average pain intensity during the last 3 days; NRS_rest_, the pain intensity at rest during the last 3 days; NRS_now_, the pain intensity at the moment during the last 3 days; NRS_unavg_, the average unpleasantness of pain during the last 3 days; NRS_unnow_, the unpleasantness of pain at the moment during the last 3 days. RMDQ, Roland-Morris Questionnaire; SAS, Self-Rating Anxiety Scale; SDS, Self-Rating Depression Scale. *p < 0.05; **p < 0.01.

### Pain intensity and unpleasantness outcomes

All data on pain intensity and the unpleasantness of pain are presented in Figure 2 and Table 2. Before the intervention, the scores of pain intensity and unpleasantness in the moderate CLBP group were higher than those in the mild CLBP group, with significant differences in the NRS_max_, NRS_avg_, and NRS_rest_ between the two groups (*p* < 0.05, Table 2). Figures 2D–F shows that the pain intensity and unpleasantness of pain scores of each group in post-intervention were lower than those in pre-intervention. A significant difference was observed in all pain intensity and the unpleasantness of pain in the overall and moderate CLBP groups and NRS_max_, NRS_avg_, NRS_now_, NRS_unavg_, and NRS_unnow_ of the mild CLBP group between pre- and post- intervention (*p* < 0.05). Compared with the mild CLBP group, the moderate CLBP group had a greater improvement, and the inter-group difference of post-pre NRS_rest_ (difference, –1.44; 95% CI: –2.69 to –0.19) was statistically significant (*p* < 0.05, Table 2). In terms of MCID, the decrease in NRS_max_ in the overall and mild CLBP groups reached MCID but not those of NRS_avg_, NRS_rest_, and NRS_now_. The decrease in NRS_max_, NRS_avg_, and NRS_rest_ in the moderate CLBP group reached MCID but not NRS_now_.

### Disability outcomes

All disability data are presented in Figure 2 and Table 2. Before the intervention, no significant difference was observed in the disability between the moderate and mild CLBP groups (*p* > 0.05, Table 2). Figures 2G–I shows that the disability of each group improved post-intervention. A significant difference was observed in the disability of the overall, moderate, and mild CLBP groups between pre- and post-intervention (*p* < 0.05). Compared with the mild CLBP group, the moderate CLBP group had more marked changes, but the inter-group difference of post-pre disability was not statistically significant (*p* > 0.05, Table 2). In addition, the decrease in RMDQ in the overall, moderate, and mild CLBP groups reached MCID.

### Negative emotion outcomes

All data of negative emotions are presented in Figure 2 and Table 2. Before the intervention, no significant difference was observed in the anxiety and depression between the moderate and mild CLBP groups (*p* > 0.05, Table 2). Figure 2J–L shows that the anxiety and depression of each group in post-intervention were alleviated. A significant difference was noticed in the negative emotions of the overall, moderate, and mild CLBP groups between pre- and post-intervention (*p* < 0.05). Compared with the mild CLBP group, the moderate CLBP group had more marked changes, but the inter-group difference of post-pre disability was not statistically significant (*p* > 0.05, Table 2).

## Discussion

This study explored the effect of an 8-week exercise intervention on social distance and CLBP symptoms in subjects with CLBP. We observed that the social distance of subjects with CLBP was shorter than that in pre-intervention. In addition, the pain intensity, unpleasantness of pain, disability, anxiety, and depression were reduced compared with those before the intervention.

### Social distance

The study showed that the subjects with CLBP shorten social distance after receiving exercise intervention. The decrease in interpersonal distance reflects the alleviation of social withdrawal and the improvement of social interaction and function. Similar findings have also been reported in other papers. A systematic review conducted by Koren et al. (2021) pointed out that Tai Chi can promote social interaction and participation and increase social support. Likewise, other exercise interventions have similar effects. Nishida et al. (2016) observed that a youth with severe social withdrawal was relieved of anxiety and depression after the treatment with sertraline alone, but social withdrawal still existed. When combined with exercise therapy, the symptoms of social withdrawal significantly improved. In addition, a high level of physical activity had been associated with a low degree of social isolation among the elderly (Robins et al., 2018). The explanation that exercise improves social withdrawal can be multifactorial. The social interaction hypothesis holds that a stable social relationship exists in sports activities. Individuals participating in sports provide support to each other, which make exercise beneficial on mental health (Peluso and Guerra de Andrade, 2005). The intervention of this study adopted the way of team training rather than one-to-one guidance, which provided subjects with more opportunities for social contact and promoted social interaction and support. Furthermore, sports activities with more social connections are more conductive to reducing social isolation and loneliness, and improving health than individual sports (Brady et al., 2020). On the other hand, the bilateral temporal hemodynamics increased significantly in the youth with social withdrawal after a 3-month jogging intervention (Nishida et al., 2016). The temporal-parietal junction belongs to the theory of mind (TOM) network which is related to prosocial actions. When the network activity increases, the shortening of social distance is one of its manifestations (Simon and Walker, 2018). Moreover, numerous studies stated that exercise can enhance the functional connection of temporal and parietal lobe and the activation of precuneus lobe, which all belongs to the TOM network. Therefore, exercise reduces social withdrawal by activating the TOM network. This series of evidence supports that exercise improves psychosocial health through neurophysiological regulation.

### Pain, disability, and negative emotions

After 8 weeks of Tai Chi intervention, the pain, disability, and negative emotions of subjects with CLBP decreased. The effect of Tai Chi on CLBP has also been confirmed in different studies. A meta-analysis of Tai Chi for LBP showed that Tai Chi can relieve pain and improve physical function (Qin et al., 2019). Hall et al. (2009) divided patients with non-specific CLBP into the Tai Chi and waitlist control groups. The Tai Chi group received Tai Chi for 10 weeks, with each session lasting for 40 min, and being conducted 1–2 times a week. The results revealed that the Tai Chi group was better than the waitlist control group in reducing the bothersomeness of back symptoms, pain intensity, and pain-related disability (Hall et al., 2011). Wang et al. (2010) conducted a meta-analysis that included 40 studies of Tai Chi on psychological health. The results suggested that Tai Chi can reduce depression, anxiety, mood disturbance, and mental pressure. Traditional Chinese medicine believes that Tai Chi improves LBP based on the interaction of “yin,” “yang,” and “qi.” It can promote the balance of “yin” and “yang” and the free flow of “qi,” which is conductive to physical and mental health (Peng, 2012). Physical activities related to LBP (such as sitting, standing, walking) are bound up with lower limb function. In Tai Chi training, the movements of continuous squatting and weight shift can improve the muscle strength and flexibility of lower limbs (Zou et al., 2019). Tai Chi can also enhance the lower limb muscle strength and posture control, maintain dynamic and static balance (Hong and Li, 2007; Hall et al., 2011). Moreover, combining Tai Chi with breathing, the movement is gentle and slow, which can reduce muscle tension and physical pain (Huston and McFarlane, 2016). Furthermore, Tai Chi can alleviate pain and disability by improving the cognition of LBP or reducing psychological pressure (Wayne and Kaptchuk, 2008; Sherman et al., 2010). Hall et al. (2011) also reported that pain-catastrophization plays the role of a mediator between Tai Chi and symptoms related to CLBP.

On the other hand, compared with the mild CLBP group, the moderate CLBP group showed more improvement in all aspects, and such result may be due to the more severe pain, disability, and negative emotions. Our previous study on CLBP and social distance revealed that pain, disability, anxiety, and depression were positively correlated with social distance (Weng et al., 2021). The improvement of CLBP was associated with a high likelihood of subsequent decrease in social distance. The amygdala is not only related to emotional processing but also a key brain region regulating social distance (Kennedy et al., 2009; Martinsen et al., 2018; Kami et al., 2020; Weng et al., 2021). Kami et al. (2020) explored the effect of voluntary running on amygdala using partial sciatic-nerve ligation model mice. They observed that voluntary running cannot only inhibit the negative emotions, such as anxiety and fear, that are closely related to the chronicity of pain, but also promote pleasure and alleviate pain: this finding might have been caused by the plasticity of the amygdala. It also provided evidence indicating that exercise can improve CLBP symptoms and adjust social distance directly or indirectly by regulating the amygdala.

### Limitations

This study had several limitations. First, only one experimental group was included. Therefore, waitlist or other intervention control groups should be considered in future research. Second, we did not perform follow-up after 8 weeks of exercise intervention. Thus, we should continue to explore the maintenance effect of exercise on the social distance of CLBP patients. Third, a narrow age range of the recruited subjects was used and there were imbalances in the gender sample size. Thus, future research designed for a stratified age and gender is needed.

### Practical implications and future prospects for research

CLBP is one of the most common chronic diseases, and causes serious harm to physiological, psychological, and social functions. However, its damage to psychosocial health is usually ignored. Patients with CLBP show increased social distance and withdrawal, reflecting the impairment of individual psychosocial health. This outcome is one of the hazards of CLBP that cannot be ignored. Through a longitudinal intervention experiment, we observed that exercise not only alleviated the symptoms related to CLBP but also improved psychosocial health. Therefore, for the management of this disease in the future, we should also pay attention to the psychological problems and consider exercise therapy as a complementary means of psychological and physiological rehabilitation. In addition, future research can be combined with neuroimaging technology to detect changes in brain activity to explain the underlying neural mechanism of exercise in improving abnormal social distance regulation behavior. It can provide a basis for CLBP to formulate more targeted intervention methods.

## Conclusion

The 8-week exercise intervention cannot only shorten the social distance and improve the abnormal behavior of social distance regulation of middle-aged and elderly patients with CLBP, but also relieve pain, disability, and negative emotions. In addition, follow-up will be conducted in the future to explore the long-term effects of exercise intervention, and further work is required to determine whether this new finding applies equally to other types of exercise.

## Data availability statement

The original contributions presented in this study are included in the article/Supplementary material, further inquiries can be directed to the corresponding author.

## Ethics statement

The studies involving human participants were reviewed and approved by the Ethics Committee of Shanghai University of Sport. The patients/participants provided their written informed consent to participate in this study.

## Author contributions

L-MW and X-QW conceived and designed the study. L-MW, RW, Q-HY, T-TC, C-CW, W-LL, S-HD, and Y-CW collected the data. L-MW and RW analyzed and interpreted the data and revised the manuscript for important intellectual content. L-MW drafted the manuscript. All authors discussed the results, commented on the manuscript, and approved the final manuscript.

## Supplementary material

Supplementary Figure 1 Flow diagram of the subjects. NRSavg, the average pain intensity during the last 3 days; CLBP, chronic low back pain.

## Abbreviations

CLBP: chronic low back pain
RMDQ: Roland-Morris Questionnaire
SAS: Self-Rating Anxiety Scale
SDS: Self-Rating Depression Scale
LBP: Low back pain
MCID: minimal clinically important difference
TOM: theory of mind (TOM).
