# Supplementary figures and images for: Effect of exercise intervention on social distance in middle-aged and elderly patients with chronic low back pain
Source: Front Aging Neurosci. 2022 Aug 22;14:976164. doi: 10.3389/fnagi.2022.976164 (PMC9441739; doi:10.3389/fnagi.2022.976164)

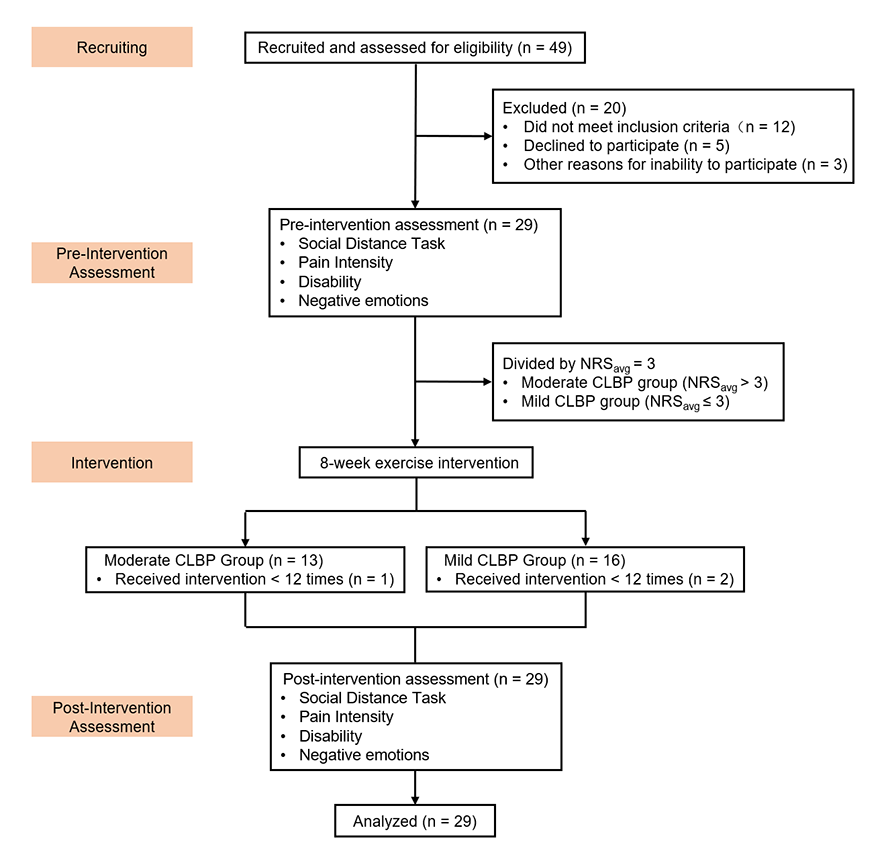

Supplement: Supplementary Figure 1 — Flow diagram of the subjects. NRSavg, the average pain intensity during the last 3 days; CLBP, chronic low back pain. [file Image_1.tif]
